# Tardive Dyskinesia With Chorea-Ballism Improved by Valbenazine: A Case Report

**DOI:** 10.7759/cureus.54666

**Published:** 2024-02-22

**Authors:** Shinichi Ichihashi, Akihiro Iha, Saori Yasumura, Shingo Kariya

**Affiliations:** 1 Division of Psychiatry, Katsuren Hospital, Itoman, JPN; 2 Division of Rehabilitation, Katsuren Hospital, Itoman, JPN; 3 Division of Nutrition, Katsuren Hospital, Itoman, JPN; 4 Division of Internal Medicine, Katsuren Hospital, Itoman, JPN

**Keywords:** vesicular monoamine transporter-2 inhibitor, antipsychotic, valbenazine, chorea-ballism, tardive dyskinesia

## Abstract

Tardive dyskinesia (TD) is an involuntary muscle movement typically caused by prolonged exposure to antipsychotic medications. Depending on the symptom severity and the affected body parts, it can cause a terrible decline in patients’ daily activities and life quality. TD often persists despite discontinuation of the offending drugs. There was no approved or effective agent to treat the patients until valbenazine, a vesicular monoamine transporter-2 inhibitor, became available. We report the case of a 64-year-old woman who started to take antipsychotics at the age of her late 20s for her schizophrenic symptoms and later developed left arm chorea-ballism in mid-50s. The patient’s involuntary movements got progressively worse even after being freed from the medications and caused severe body weight loss due to difficulties in taking meals. Daily treatment with valbenazine gradually mitigated her symptoms, resulting in significant improvement in her feeding activities, body weight, and daily life quality. This is the first report, to our knowledge, describing the therapeutic potential of valbenazine to improve chorea-ballism associated with TD. Our observation highlights that valbenazine may relieve a broader spectrum of antipsychotic-induced involuntary movements.

## Introduction

Tardive dyskinesia (TD), an iatrogenic brain disorder characterized by a wide range of hyperkinetic involuntary movements, is primarily caused by the long-term use of dopamine-receptor-blocking agents, such as antipsychotic medications [1]. The prevalence of TD is approximately 25% among patients receiving first- or second-generation antipsychotics [2]. TD typically occurs as a mixture of facial grimacing, flicks of the tongue, and smacking of the lips in the oral-buccal-lingual region, which, in serious cases, leads to difficulties in speaking, swallowing, and eating [1]. TD also frequently appears in the distal limbs as a form of chorea, which is relatively larger in movements like a piano-playing motion of the fingers [3]. Depending on the symptom severity and the affected body parts, TD can cause a terrible decline in patients’ daily activities and life quality.

The symptoms of TD often persist and even worsen despite switching medication to less potent dopamine antagonists or discontinuation of the offending drugs. There was no approved or effective agent to treat the patients until valbenazine, a vesicular monoamine transporter-2 (VMAT2) inhibitor, became available [4]. In TD, excessive responses of postsynaptic D2 receptors, which have become abnormally sensitive due to chronic exposure to dopamine-receptor blockers, are thought to be associated with the emergence of involuntary movements [5]. The mechanism of action of VMT2 inhibitors is to reduce dopamine availability at synaptic clefts, thereby suppressing the binding of dopamine to the D2 receptors [5].

Ballism produces flinging movements of high amplitude and appears more proximal in location as compared to chorea [6]. It is a less common manifestation in TD [3]. We report here a rare case of a 64-year-old woman with TD-associated chorea-ballism, which was successfully treated by valbenazine.

## Case presentation

The patient is a 64-year-old woman, who worked as a kindergarten teacher after graduating from a junior college and has no particular past illness or family history to be noted. She started to experience auditory hallucinations and suicidal ideation at the age of her late 20s and was diagnosed as having schizophrenia. Thereafter, she had been repeatedly in and out of the psychiatric hospitals and treated for more than three decades with several kinds of classical antipsychotics (details unknown). The patient started to develop irregular and rapid involuntary movements, such as eye squinting and chewing-like motions around the 40s. Later in the mid-50s, she further developed left-hand choreiform movements like playing the piano and a ballistic left-arm shaking movement. While these involuntary movements, suggesting TD, had slowly intensified over the years, she received no particular treatment for the symptoms. At the age of 63, she committed suicide by jumping from her home balcony, resulting in a bedridden state due to bilateral pneumothorax and complex fractures in her ribs, pelvis, and dominant right arm. Since then, she had to take meals with her non-dominant left arm despite the chorea-ballism. A year later, the patient was transferred to the long-term care unit of our hospital for further supportive care.

**Figure 1:**
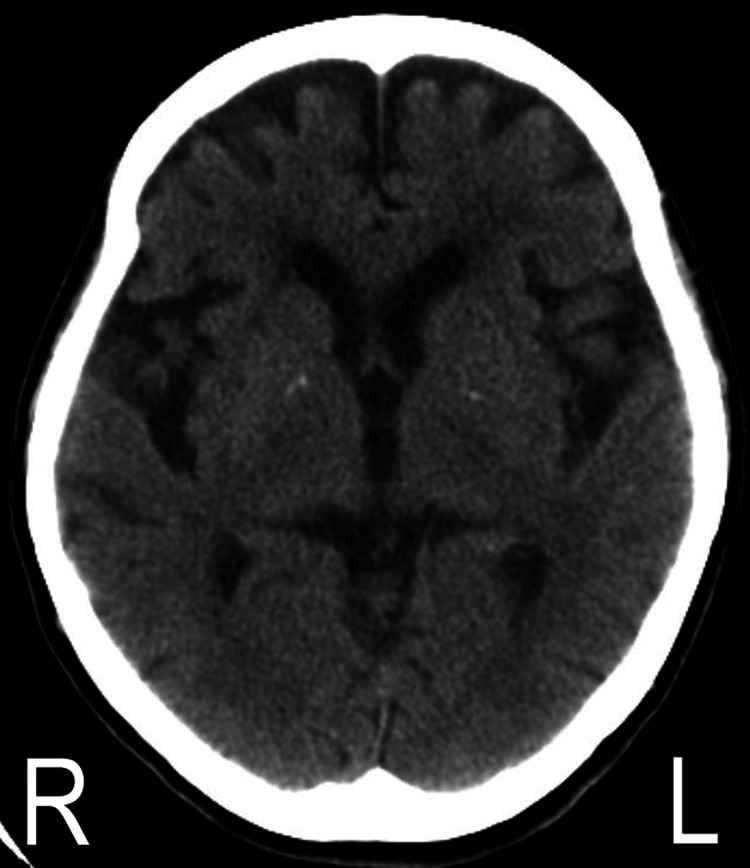
Brain CT scan on admission An axial image at the level of basal ganglia shows bilateral mild frontotemporal atrophy.

On admission (day 1), the patient had no suicidal ideation or hallucination of any kind but was in a state of residual schizophrenia manifesting avolition, anhedonia, and asociality. She preferred to stay in bed all day except for her three mealtimes. Physically, she was skinny (body weight: 39.9 kg, height: 155 cm) with a low body mass index (BMI: 16.6). The patient had mild muscle atrophy and weakness in her trunk and limbs due to the chronic bed rest with only minimum physical activity. Neurological examinations revealed normal deep-tendon reflexes and muscle tonus in her extremities and neither sensory defect, pathologic reflex nor ataxia was presented. The TD-associated typical movements in her orofacial region were not that serious to cause dysphagia, abnormal speech, or self-biting. In contrast, her left arm chorea-ballism, particularly the ballistic movement like a repetitive (~1Hz) saluting motion (Video 1), prevented the patient from taking meals in a relaxed manner and caused excessive food spills. The unavailability of her dominant right arm, which has been disabled since the previous fractures, made the patient’s feeding activity further difficult. The chorea in her left hand, which had been like a piano playing motion at the time of onset in her 50s, exhibited a more amplified movement like a repetitive milking motion. Her left arm ballism repeated transient waning every minute or so, and she took meals in a rush when her arm shaking was relatively calm. All of her involuntary movements were not diminished by distraction and were completely absent during sleep. The total score of the Abnormal Involuntary Movement Scale (AIMS) was 17 [7]. No notable abnormality was detected in the patient’s blood test, including blood sugar and thyroid hormones. There was no unusual finding in her brain computed tomography (CT) except for bilateral mild frontotemporal atrophy (Figure 1), while a magnetic resonance image (MRI) scan could not be performed due to orthopedic metal implants in her body.

**Figure 2:**
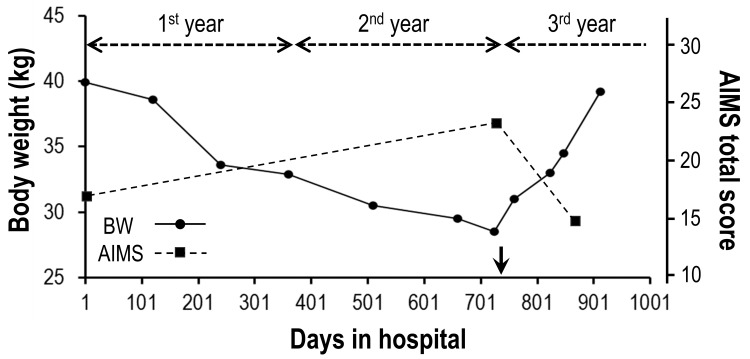
Temporal change of body weight and AIMS total score after hospitalization The body weight (BW) declined constantly from 39.9 kg (day 1) to 28.5 kg (day 725) during the first two years of hospitalization. On day 730 (arrow), the daily treatment with valbenazine was initiated and the patient started to regain her BW (day 913: 39.2 kg). The AIMS total score, which was 17 on day 1, increased to 23 on day 725 but improved to 15 (day 867) after the treatment with valbenazine. AIMS - Abnormal Involuntary Movement Scale

As the patient was free from positive symptoms of schizophrenia, we first discontinued 0.75 mg of haloperidol, the prototypical first-generation antipsychotic, that she had been taking daily for more than a few decades. While the patient’s mental state remained stable during a year of observation without any antipsychotics, her body weight declined further (Figure 2) due to no improvement in her left arm symptoms. To manage the patient’s involuntary movements ruining her health and life quality, we next tried an off-labeled agent, clonazepam, reported to be effective in some TD cases [8,9]. Several doses (0.5-1.5 mg/day) were tested; however, clonazepam only caused sluggishness, resulting in its discontinuation. Note that, even if the patient’s emaciation becomes life-threatening, neither tube feeding, intravenous nutrition, surgical interventions (e.g., pallidotomy, deep brain stimulation) nor electroconvulsive therapy was an alternative for our patient, respecting the wishes of the patient and her relatives, which we had confirmed on several occasions. During the second year, the patient’s involuntary movements got gradually worse, and her body weight loss further progressed (Figure 2). On day 725, the AIMS total score was 23 and the body weight was 28.5 kg (BMI: 11.9).

**Video 1:** Videography taken on the first day of treatment with valbenazine In addition to the mild orofacial dyskinesia (not visible due to artificial masking), apparently rough and wide-amplitude involuntary movements (~1Hz), considered as chorea-ballism, are recognized exclusively in the patient’s left upper limb. The chorea-ballism is composed of choreiform milking motions in her hand and ballistic saluting motions in her proximal upper limb.

**Figure 3:**
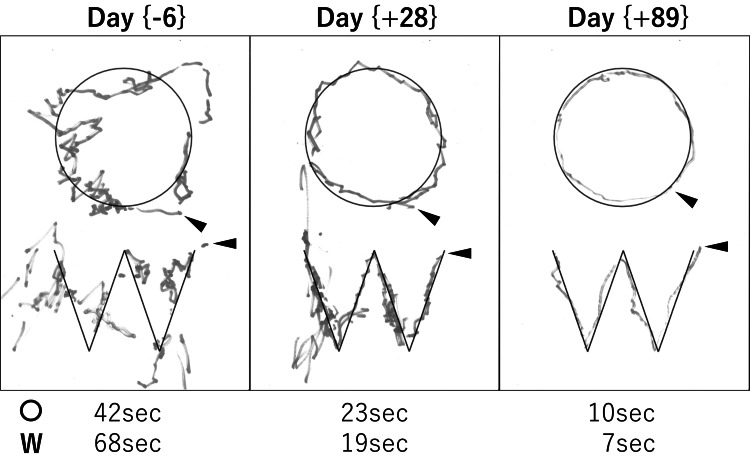
Temporal change of tracing skill The patient was asked to trace along given shapes (○ and W printed on an A-four size paper) with a red marker (the figure is converted into black and white) in her left hand. The examination was performed on days (-6), (+28) and (+89) of the daily treatment with valbenazine. Arrowheads indicate the tracing start points. Times needed to complete the tracing tasks were indicated on the bottom of each examination.

After two years (day 730) of hospitalization, we started to treat the patient with 40 mg/day of valbenazine, which has recently become available in our hospital (day X of valbenazine treatment is expressed as day (±X) for the rest). After confirming no adverse effect, a dose of valbenazine was increased to 80 mg/day on the day (+19). From about day (+25), the patient started to manifest less orofacial dyskinesia, and her left arm symptoms began to show gradual improvement (Video 2), which was well-reflected on the tracing tests done with her left hand (Figure 3). On the day (-6), she could barely trace the shapes due to severe involuntary movements and required lots of time (> 30 seconds) and effort, which appeared as her strong pen pressure, to complete each shape. After starting valbenazine, such features gradually faded, showing much better tracing skills in both time and accuracy on the day (+89). Importantly, the waning of chorea-ballism enabled the patient to take meals slowly under relaxed conditions with less food spill, resulting in her body weight regain (Figure 2). The patient’s body weight recovered to 39.2 kg (BMI: 16.3) on day (+183) and the AIMS total score improved to 15 on day (+137). Of significance, the patient also started to smile more frequently and asked to be seated on her bed or wheelchair more often in the daytime, suggesting her overall better condition. The score of the Mini-Mental State Examination (MMSE), which could not have been done on admission due to her apathetic state of mind, was 22/30 on day (+210), suggesting her mild cognitive impairment.

**Video 2:** Videography taken on day 57 of the daily treatment with valbenazine The patient’s orofacial dyskinesia (not visible due to artificial masking) and ballistic left arm motions are significantly diminished. Only a slight piano playing-like motion is recognized in her left fingers.

## Discussion

We presented the rare case of a 64-year-old woman who developed antipsychotic-induced left arm chorea-ballism, which caused severe emaciation due to difficulties in taking meals but was later treated successfully by valbenazine, leading to significant improvement in her feeding activities, body weight, and daily life quality. To our knowledge, this is the first report to describe the therapeutic potential of valbenazine to improve chorea-ballism associated with TD.

The boundaries between chorea and ballism are often blurred and, in many patients, they frequently co-appear in one-half of the body as hemichorea-hemiballism (HCHB) [10,11]. These two involuntary movements are viewed as part of the clinical spectrum of the same pathological process primarily associated with a lesion or functional impairment of the contralateral striatum or subthalamic nucleus, which in turn hyperactivates dopaminergic neurons, thereby mediates choreiform and/or ballistic movements [10,11]. In adults, HCHB can appear in various disorders, such as stroke, type 2 diabetes mellitus, autoimmune diseases, and hyperthyroidism [6,12,13]. Although MRI for the detailed brain examination was unperformable in our patient, we have no doubt regarding the diagnosis of TD, which we made based on her typical clinical history and course TD, unremarkable findings in the laboratory tests, brain CT images showing no lesion in the basal ganglia and family history without any health issues. However, the emergence of ballism is relatively rare in TD [3]. Even more unusual, the chorea-ballism in the patient was observed exclusively in her left arm for more than a decade since the onset. As an explanation for such a unique manifestation presented as “monochorea-monoballism,” possible involvement of other pathologies (e.g., poor blood perfusion or tiny infarction in the region responsible for the development of left arm hyperkinesia) cannot be denied. In our case, chronic exposure to antipsychotics may have triggered the emergence of chorea-ballism only in her left arm under the pre-existence of such minor damage, which does not cause symptoms by itself but creates regional vulnerability. Examination by positron emission tomography-CT may help us understand more precisely the patient’s disease pathology.

Clinical symptoms of patients with schizophrenia affected by TD can be quite similar to those of Huntington's disease (HD), an autosomal-dominant neurodegenerative disease characterized by hyperkinetic involuntary movement, cognitive impairment, as well as by psychosis, and emotional deterioration [14,15]. In our patient, schizophrenic hallucination and delusion appeared at the age of late 20s, followed by left arm chorea-ballism in her mid-50s. However, the psychiatric symptoms became residual in the mid-60s, and the left arm symptom never spread to other parts of her body for more than a decade until now. These clinical presentations did not match with a mercilessly progressive pattern of HD, which results in a severely disabled condition, both physically and intellectually, within a decade or so after clinical onset. We therefore concluded that our case was not HD.

Chorea-ballism often resembles and needs to differentiate from a stereotypy, which was defined by Edwards et al. as “a non-goal-directed movement pattern or vocalization that is repeated continuously for a period of time in the same form and on multiple occasions, and which is typically distractible” [16]. Adult-onset motor stereotypies can appear in various neuropsychiatric conditions including schizophrenia, excessive dopaminergic treatment of Parkinson's disease, degenerative dementias, and exposure to psychostimulants, such as cocaine [17-19]. Striatum abnormalities are considered responsible for the development of stereotypic behaviors [19]. A recent study conducted by Baizabal-Carvallo and Jankovic identified that a cohort of patients with stereotypy manifests sudden onset (84%), prominent distractibility (58%), and periods of unexplained improvement (84%) that were not reported in patients with TD [20]. Such features were also not seen in our case. Thus, the left arm movement that appeared in our patient was considered as chorea-ballism associated with TD. 

As for a condition responsible for the cerebral atrophy detected in the CT scan, we suspected the possibility of behavioral variant frontotemporal dementia (bvFTD), which is characterized by a progressive decline in social functioning and changes in personality with an average onset around the early 50s [21]. Provided that the psychiatric symptoms in our patient are partially associated with bvFTD, she can be diagnosed, together with her mild cognitive impairment and frontotemporal atrophy (Figure 1), as probable bvFTD classified in the international consensus criteria [22]. Importantly, growing evidence demonstrates shared genetic and pathoanatomical links between FTD and schizophrenia, indicating potential common molecular mechanisms contributing to their overlapping pathophysiological and clinical characteristics [23-25].

Given the effect of VMAT2 inhibitors to reduce dopamine availability at synaptic clefts, it is possible that chronic administration of valbenazine may eventually mediate an altered balance of dopamine-related neural homeostasis, such as downregulation or hyposensitivity of postsynaptic D2 receptors, which would potentially lead to the development of Parkinsonian syndromes. In fact, there are some reports describing the valbenazine-induced parkinsonism [26,27]. As regards our patient, we will continue the current pharmacotherapy for the time being, while carefully monitoring for its common side effects (e.g., problems in vision, urination, and cardiac rhythm) and possible tardive adverse effects.

## Conclusions

We reported that chorea-ballism, which appeared as an atypical manifestation of TD and caused serious decline of health and life quality in our patient, can be effectively moderated by valbenazine, the VMAT2 inhibitor available now to treat TD. This case report highlights that valbenazine may relieve a broader spectrum of antipsychotic-induced involuntary movements.
